# Off Starburst Amacrine Cells in the Retina Trigger Looming-Evoked Fear Responses in Mice

**DOI:** 10.1523/ENEURO.0183-22.2023

**Published:** 2023-04-10

**Authors:** Jeremy M. Bohl, Jui Gope, Zachary J. Sharpe, Angela Shehu, Andrew Garrett, Christina C. Koehler, Chase B. Hellmer, Tomomi Ichinose

**Affiliations:** 1 Department of Ophthalmology, Visual and Anatomical Sciences, Wayne State University School of Medicine, Detroit, MI 48201; 2 Department of Public Health, Wayne State University School of Medicine, Detroit, MI 48201; 3 Department of Pharmacology, Wayne State University School of Medicine, Detroit, MI 48201

**Keywords:** fear response, flight, looming stimulus, starburst amacrine cell, vision test

## Abstract

A rapidly approaching dark object evokes an evolutionarily conserved fear response in both vertebrates and invertebrates, young to old. A looming visual stimulus mimics an approaching object and triggers a similarly robust fear response in mice, resulting in freeze and flight. However, the retinal neural pathway responsible for this innate response has not been fully understood. We first explored a variety of visual stimuli that reliably induced these innate responses, and found that a looming stimulus with 2-d acclimation consistently evoked fear responses. Because the fear responses were triggered by the looming stimulus with moving edges, but not by a screen flipping from light to dark, we targeted the starburst amacrine cells (SACs), crucial neurons for retinal motion detection. We used intraocular injection of diphtheria toxin (DT) in mutant mice expressing diphtheria toxin receptors (DTR) in SACs. The looming-evoked fear responses disappeared in half of the DT-injected mice, and the other mice still exhibited the fear responses. The optomotor responses (OMRs) were reduced or eliminated, which occurred independent of the disappearance of the fear responses. A histologic examination revealed that ON SACs were reduced in both mouse groups preserved or absent fear responses. In contrast, the number of OFF SACs was different among two groups. The OFF SACs were relatively preserved in mice exhibiting continued fear responses, whereas they were ablated in mice lacking fear response to looming stimulation. These results indicate that OFF SACs and the direction-selective pathway in the retina play a role in looming-induced fear behaviors.

## Significance Statement

In response to a suddenly approaching dark object, mice exhibit a defensive response: either flight to a refuge or freeze in the same spot. How the visual system evokes this response in the mouse has not been fully understood. We focused on the initial neural component of the visual system, the retina, and examined a type of neuron known for sensing object motion: the starburst amacrine cell (SAC). We found that ablation of OFF starburst amacrine cells removed the dark object-evoked defensive responses. We believe that our findings will contribute to understanding the neural network connecting the visual system and the brain circuit regarding fear and emotion.

## Introduction

The fear response to an approaching predator is a crucial innate behavior for survival. The looming object-evoked fear response occurs in many species of vertebrates and invertebrates that are equipped with visual systems, including locusts, flies, fish, rodents, and humans from infancy to adulthood (Schiff et al., 1962; Blanchard et al., 2001, 2002; Yilmaz and Meister, 2013; Temizer et al., 2015; Klapoetke et al., 2017; Bertels et al., 2020). The looming stimulus consists of an expanding dark circle above the head that robustly induces fear responses in mice (Yilmaz and Meister, 2013; De Franceschi et al., 2016; Koehler et al., 2019). Flight (dash to a refuge) or freezing is commonly observed in response to the looming stimulus, although a diversity of responses among mice is documented (Yilmaz and Meister, 2013; De Franceschi et al., 2016; X. Yang et al., 2020).

Neural networks responsible for the looming behavior have been investigated in the central nervous system (Mobbs et al., 2009; J. Yang et al., 2012; Ren and Tao, 2020); however, looming stimulus-sensing retinal neural circuits have not been fully understood. In the mouse retina, visual signals are captured by photoreceptors and transferred to ∼15 types of bipolar cells (Euler et al., 2014; Ichinose et al., 2014; Ichinose and Hellmer, 2016; Shekhar et al., 2016), which relay differing visual features to 60 types of amacrine cells (Yan et al., 2020) and 50 types of ganglion cells (Baden et al., 2016). Most retinal neurons can be further categorized as ON or OFF types, which respond to light or dark objects, respectively. Because an expanding dark spot with moving edges uniquely evokes fear responses, the OFF channel of direction-selective cells in the retina are expected to contribute predominantly to looming-evoked behavior (Yilmaz and Meister, 2013).

Two types of OFF cell pathways have been shown to convey looming-evoked fear responses. The PV-5 OFF ganglion cells are sensitive to approaching motion (Münch et al., 2009). Also, a neural pathway of vGluT3 amacrine cells, W3 and OFF t-α ganglion cells mediate the looming-evoked defensive responses (Kim et al., 2020; Wang et al., 2021). The latter pathway is critical for sensing local motion, and the ablation of these neurons eliminates the defensive responses to looming stimuli (Kim et al., 2020; Wang et al., 2021). However, there might be other mechanisms in the retinal network, similar to those found in the fly and locust visual system (Gabbiani et al., 2002; Klapoetke et al., 2017). The direction-selective pathway (Yoshida et al., 2001; Euler et al., 2002), may sense the looming stimulus as a dark moving edge.

To investigate whether the direction-selective pathway in the retina contributes to looming detection, we ablated starburst amacrine cells (SACs) using an intraocular injection of diphtheria toxin (DT). Looming stimulus-evoked behaviors and the optomotor responses (OMRs) were monitored before and after the SAC ablation. We found that OFF SACs and the direction-selective pathway are critical components for looming-induced fear responses.

## Materials and Methods

### Ethical approval

All animal purchasing, handling and procedures were approved by the Institutional Animal Care and Use Committee at Wayne State University School of Medicine. All the necessary steps were taken to minimize animal suffering and all experimentation was conducted in accordance with relevant guidelines and regulations. The tissues were harvested immediately after the animals were euthanized by CO_2_ inhalation and cervical dislocation.

### Animals

Transgenic mice expressing Cre recombinase in SACs (ChAT-Cre, stock #031661, The Jackson Laboratory, RRID: IMSR_JAX:031661) were crossed with a mouse line containing the diphtheria toxin receptor, the expression of which can be induced by Cre- mediated recombination (ROSA-iDTR, stock #007900, The Jackson Laboratory, RRID: IMSR_JAX:007900). Control mice were of the C57BL/6J strain (stock #000664, The Jackson Laboratory, RRID: IMSR_JAX:000664). Mice were maintained in group cages separated by sex; both males and females were used in the study. Mice were provided with food and water *ad libitum* and maintained on a 12/12 h light/dark cycle.

### Visual testing

The methods of visual stimulation and mouse handling were based on the procedures described previously (Yilmaz and Meister, 2013; De Franceschi et al., 2016; Koehler et al., 2019). In brief, the testing arena consisted of a 40 × 50 × 33 cm (width × length × height) enclosure with an overhead computer monitor for stimulus display and a plastic hut to serve as a shelter. The arena was held at a mesopic light level of 7 × 10^5^ photons/μm^2^/s before visual stimulation. Stimuli were generated in MATLAB (MathWorks, RRID: SCR_001622) using PsychToolbox3 (RRID: SCR_002881). The day before testing, each mouse was given 10 min to acclimate to the testing arena and become familiar with the location of the shelter. On testing day, mice were given another 10 min to acclimate to the arena before stimulus onset. When a mouse was at least 500 mm from the shelter, a visual stimulus was presented. The mouse’s movement during each trial was video captured using a CCD camera (Ultrasensitive CCD, Teledyne Lumenera) with associated recording software (Stream Pix 7, NorPix, RRID: SCR_015773). Each mouse received only one trial of the looming stimulus in a given day.

**Figure 1.**
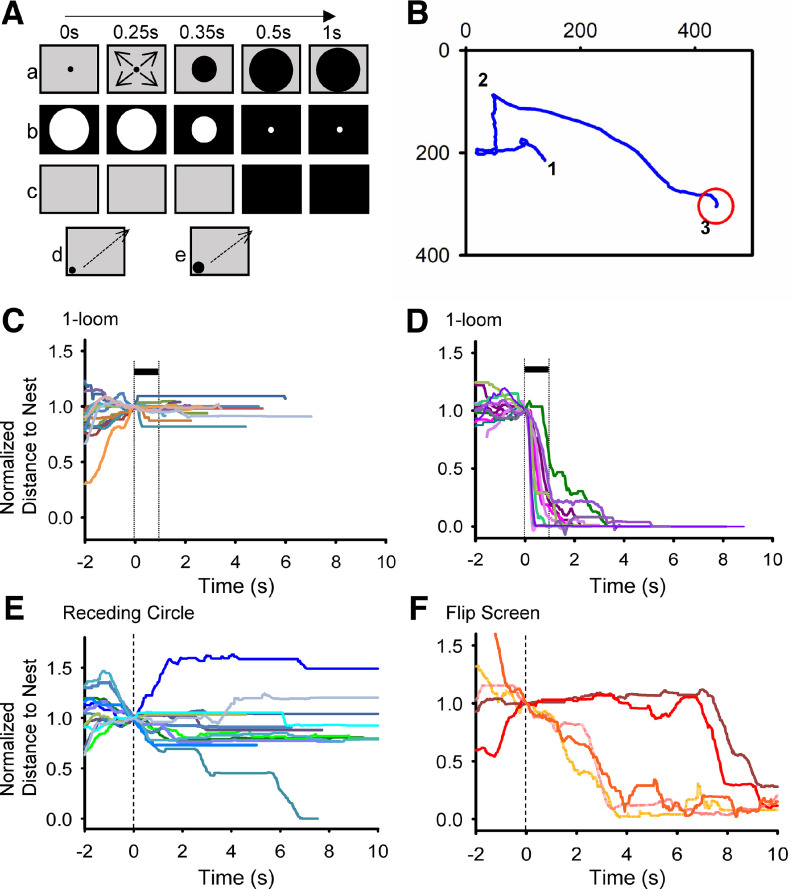
Visual stimuli and mouse behavior. ***A***, Visual stimuli we tested: ***a***, looming stimulus; ***b***, white receding stimulus; ***c***, flip screen stimulus; ***d***, sweeping stimulus; ***e***, large sweeping stimulus. ***B***, A sample mouse track record. A mouse was placed in an arena of 400 × 500 mm with a hut for the mouse to escape from the stimulus (red circle). After 10-min acclimation, video capturing started (1), a looming stimulus started (2), and the mouse came into the shelter (3). ***C***, Based on the mouse tracking records, the distance to the nest over the time was calculated and plotted. After normalization to the distance at the stimulus onset (time = 0 s), the distances for individual mice were plotted before and after the 1-time looming stimuli. A dark bar indicates the timing of the 1-looming stimulus. The vertical dashed line at time 0 s indicates stimulus onset. The 1.0 indicates the position when the visual stimulus started, while the location of the shelter was 0. A group of mice froze to the stimuli (14/26 mice). Each line color indicates the track of each mouse. ***D***, The same stimulus evoked immediate flight responses to another group of mice (12/26 mice). ***E***, 10-time receding white circle evoked freeze responses in 13/25 mice. ***F***, Flip screen did not evoke fear responses (6 mice).

Each mouse was tested with one of the three stimuli: looming, receding circle, or flip screen. The primary looming stimulus was a 2° black disk that rapidly expanded until it reached 50° within 250 ms on a gray background (Fig. 1*Aa*). In some cases, the looming stimulus expanded from 2° to 9°. Mice were tested with either a single looming stimulus or a looming stimulus that repeated 10 times (1-time or 10-time). The receding white circle stimulus consisted of a large white circle receding to a small dot on a black background (Fig. 1*Ab*). The flip screen stimulus changed from a gray background to a dark background instantaneously (Fig. 1*Ac*). The sweeping circle stimulus (De Franceschi et al., 2016) was used to simulate a predator searching for prey. Two sizes were used: a smaller black disk that made a 5.7° visual angle size to a mouse in the arena, and a larger 8.5° black disk, both at a speed of 21° per second (Fig. 1*Ad*,*e*).

### Optomotor response (OMR) and slow-angled descent forepaw grasping (SLAG) test

Optomotor response (OMR) testing was used to assess each mouse’s visual contrast sensitivity before and after diphtheria toxin injection using the Optodrum system (Striatech). The walls of the square testing arena consist of four computer screens In the center of the arena, a mouse is placed on an elevated stage. The mouse sits unrestricted on the stage as the walls project a rotating black and white striped stimulus that triggers the OMR. A camera at the top of the Optodrum tracks the mouse’s head movements. The contrast sensitivity was measured at a spatial frequency of 0.064 cycles/degree. The presence or absence of optomotor tracking was automatically tracked by the included software package which analyzes the correlation between the angular velocities of the mouse’s head and the rotation of the stimulus. Mice were tested at a variety of contrast levels set automatically by the software’s staircase algorithm, which was provided by the system (Striatech), and used by Prusky et al. (2004), which determined the step size between contrast levels. Clockwise and counterclockwise stimuli were used to determine optomotor reflexes driven by the left and right eyes, respectively (Prusky et al., 2004; Douglas et al., 2005). Testing of a combined control/experimental group was performed using a single blind method where groups were mixed together and referred to by a temporary identifier with the condition unknown to the experimenter. Contrast thresholds obtained in OMR testing were converted to contrast sensitivities by calculating their reciprocal.

Mice were tested for functional vision after DT injection using the slow-angled descent forepaw grasping (SLAG) test (Gil-Pages et al., 2013). Mice held by the tail were lowered over a wire cage lid until they were several centimeters away from the edge. A mouse with functional vision will reach toward the cage in an attempt to grasp it. The procedure is repeated with the mouse facing away from the lid of the cage, and a mouse with functional vision will twist to reach toward the lid. Videos were recorded and scored by two experimenters.

### Intraocular diphtheria toxin injection

Procedures regarding the diphtheria toxin injection timing and concentration followed the method previously described (Hillier et al., 2017; Kim et al., 2020). Mice were anesthetized for intraocular injection using a mixture of ketamine (80 μg/kg) and xylazine (5 μg/kg). Glass micropipettes for injection were prepared using a P1000 micropipette puller (Sutter Instruments). Injection was performed using a Nanoject III precision pipette (Drummond Scientific). DT (D0564, Millipore Sigma) was diluted to a working concentration of 0.8 ng/μl, and 2 μl was injected into the intravitreal space of each eye twice, 48 h apart. For some mice, 2 μl DT solution at a concentration of 5 ng/μl was injected to the intravitreal space only once. Subsequent behavioral and cellular experiments were conducted 6–10 d after the initial DT injection. Control mice were injected with 0.9% normal saline in both eyes on the same schedule as toxin-injected mice.

### Immunostaining and imaging

Detailed methods for the mouse dissection and our standard immunostaining procedures are described in our previous publication (Ichinose et al., 2014; Farshi et al., 2016). In brief, eyes were enucleated immediately after euthanasia with CO_2_ gas and cervical dislocation, and were transferred to a dissection chamber with oxygenated HEPES buffer solution composed of the following (in mm): 115 NaCl, 2.5 KCl, 2.5 CaCl_2_, 1.0 MgCl_2_,10 HEPES, and 28 glucose, adjusted to pH 7.37 with NaOH. Retinal tissue was isolated, and mounted on a piece of nitrocellulose filter paper. Retinal tissue on the filter paper was fixed for 60 min in 4% paraformaldehyde in 0.1 m phosphate buffer (PB). Tissue was blocked for 60 min in 10% normal donkey serum (NDS) dissolved in 0.5% Triton X-100 in 0.01 m PBS (PBS-T). Primary antibody goat anti-choline acetyltransferase (ChAT-antibody, Millipore Sigma catalog #AB144P, RRID: AB_2079751) at 1:200 concentration was dissolved in 3% NDS in PBS-T and incubated overnight at room temperature. The secondary antibody was Donkey Anti-Goat Alexa 568 (Thermo Fisher Scientific catalog #A-11 057, RRID: AB_2534104) or Donkey Anti-Goat Alexa 633 (Invitrogen catalog #A31083, RRID: AB_2535739) at 1:500 concentration for 2 h; 1 μm 4′,6′-diamino-2-phenylindole (DAPI) in 3% NDS in PBS-T was added for 15 min at room temperature. Tissue was washed and mounted for imaging with a confocal microscope (TCS SP8, Leica).

### Data analysis and statistics

Video files generated during stimulus testing were analyzed as follows; the location of the mouse’s nose in each frame was manually tracked using software (Image Pro Plus, RRID: SCR_016879, Media Cybernetics), which returned the *x* and *y* coordinates in the arena versus time. The video images were skewed, which were corrected, accordingly (Koehler et al., 2019). The speed of the mouse’s movement was calculated based on the *x-y* location differences between two frames. The maximum speed was the highest speed measured during the actual flight or escape response.

Mouse response to a visual stimulus was separated into three categories based on previous publications (De Franceschi et al., 2016; Koehler et al., 2019). Flight: a mouse dashed to the shelter with the following three criteria: (1) the speed was at least 300 mm/s; (2) the flight speed reached its maximum within 0.5 s; and (3) the mouse directly went into the nest or next to it after the initial flight response. Freeze: a mouse remained immobilized for >1 s at a speed of 0 mm/s, accompanied by no movement of the head or tail. Rearing: a mouse stood up on hind legs and observed the visual stimulus.

Cell counting was conducted using peripheral retinal tissue. One randomly selected 290 × 290 μm field per tissue with step size of 1 μm was captured to image DAPI stained cells in the ganglion cell layer (GCL). Alexa-633-labeled ON and OFF SACs were similarly imaged in the GCL and inner nuclear layer (INL), respectively. Confocal image stacks were subjected to three-dimensional deconvolution using the AutoQuant X3 platform (Media Cybernetics, RRID: SCR_002465). Cell counting of SACs was performed manually using both a cell counter plugin available with the Fiji distribution of the NIH ImageJ platform (Schindelin et al., 2012; RRID: SCR_002285) or a manual cell counter overlay available in the aforementioned LAS X software. Counting of the much more numerous DAPI-stained cells was performed using an AIVIA3D Object Analysis-Meshes recipe from Leica and DRVISION (Bellevue).

Statistical analysis was performed in Microsoft Excel (Microsoft; RRID: SCR_016137) and GraphPad Prism (GraphPad Software; RRID: SCR_002798). Either an unpaired Student’s *t* test or one-way ANOVA with *post hoc* analysis was used to compare multiple groups.

## Results

The looming stimulus evokes fear responses in mice, including freeze and flight (Yilmaz and Meister, 2013; De Franceschi et al., 2016). Cellular and molecular mechanisms for looming stimulus detection and fear responses have not been fully understood. In the current study, we focused on the direction-selective pathway in the retina to explore its contribution to visually-evoked fear behaviors.

### Wild-type mice exhibited fear responses to visual stimuli

A C57 wild-type mouse was placed in a behavior arena, and a looming stimulus was activated on a monitor placed on the ceiling of the arena (Fig. 1*Aa*; Koehler et al., 2019). The mouse was allowed to move freely in the arena (Fig. 1*B* point 1 to 2). A visual stimulus was initiated when a mouse was located in the middle of the arena and away from the shelter (red circle). If a looming stimulus was projected, the mouse quickly moved into the shelter, displaying a flight response (Fig. 1*B* point 2 to 3). The mouse’s movement was tracked and the distance to the shelter from each point was plotted as a function of time before and after visual stimulation. Figure 1*C–F* shows the distance from the nest after being normalized to position at stimulus onset. Each mouse was acclimated a day before and on the day of the looming experiment, which induced robust fear responses during the looming stimulus (see later for more detail).

**Figure 2.**
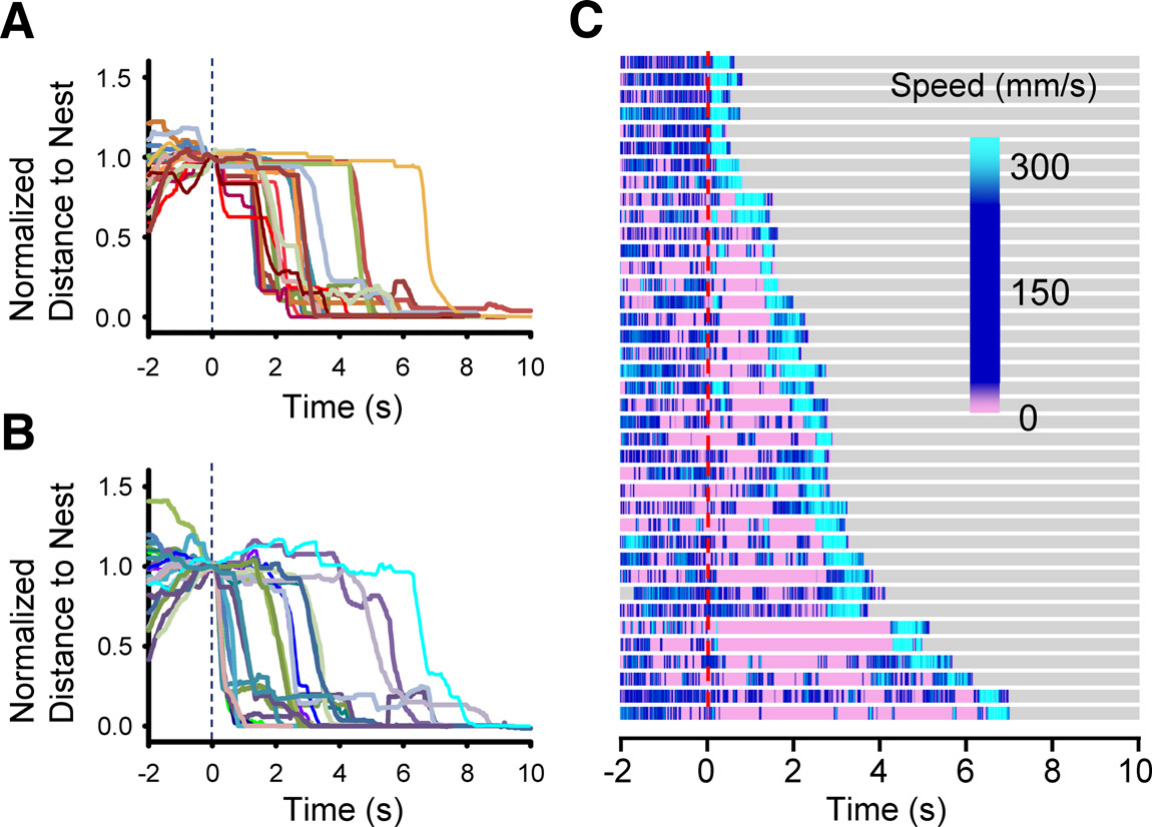
C57 wild-type mice exposed to the 10-looming stimulus. ***A***, A group of mice froze before the flight response, indicated by straight lines after the stimulus (18/39 mice). ***B***, Another group of mice exhibited immediate flight or rearing and flight responses (21/39 mice). ***C***, A heatmap summary of mouse speed before and after 10-looming stimuli. A red dashed line indicates the onset of the looming stimulus. A high speed (light blue) at the end shows the flight response before moving into a hut. A low speed (pink) for >1 s indicates the freeze response. Tracking was ended after mice moved into the hut.

**Figure 3.**
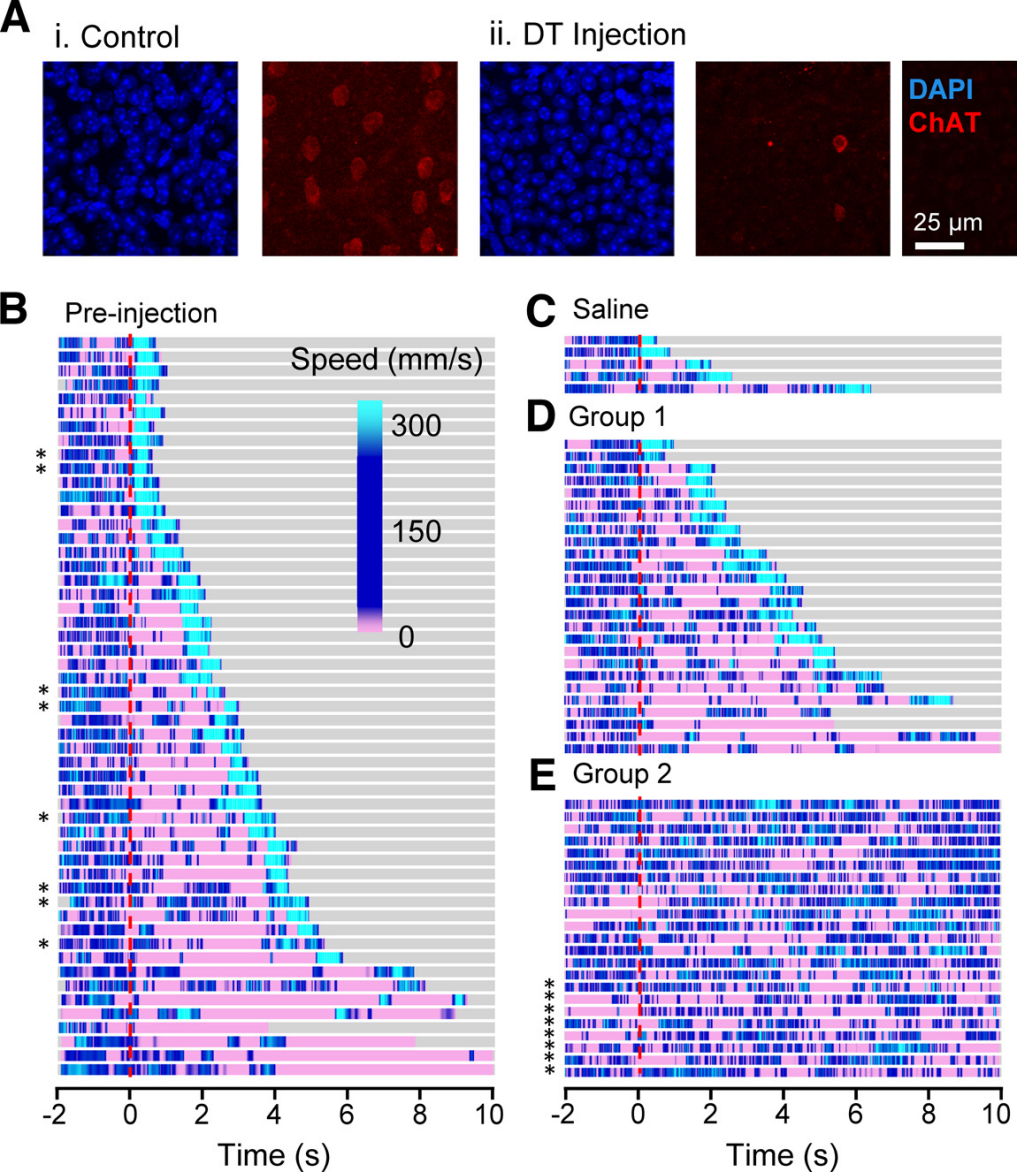
DT injection reduced the number of SACs and removed the looming-evoked fear responses. ***A***, Using immunohistochemistry, (I) control and (II) DT-injected retinas were labeled with DAPI and ChAT-antibody. There was no difference in the density of GCL layer cells between the control and injected groups. In contrast, the SAC density showed a significant reduction. ***B***, Speed heatmap for mouse responses to the 10-time looming stimulus before DT injection. After the looming stimuli, mice displayed a combination of flight (high speed, light blue) and freezing (low speed, pink) responses. A red line indicates the onset of looming stimulus. Asterisks (*) indicate that mice were exposed to the 2° to 9° looming stimulus. All other mice were exposed to the looming stimulus of 2° to 50°. ***C***, Saline-injected mice showed flight responses to looming stimulus. ***D***, Group 1 mice exhibited freeze and/or flight responses after DT injection. ***E***, Group 2 mice did not show freeze and/or flight responses after DT injection.

We evaluated the impact of several stimulus parameters on the evocation of fear responses. The one-time looming stimulus evoked either a freeze (Fig. 1*C*) or flight response (Fig. 1*D*). The probability of displaying either response was about half (Table 1). We also aimed to examine whether the direction of the darkening is crucial. We presented a group of mice with a white receding circle, which darkened the monitor from the periphery instead of the center (Fig. 1*Ab*). The white receding circle evoked freeze responses in about half of the mice we tested (Table 1; Fig. 1*E*). Mice also exhibited either flight (8%), rearing (52%), or a combination of these. The rearing response indicates weak threat-elicited behavior (X. Yang et al., 2020), thus, the white receding circle is considered to be a weak threat stimulus.

**Table 1:** The various visual stimuli-evoked behavioral responses in C57 wild-type (WT) mice

C57 WT	Mouse number	Flight	Freeze	Rearing	No response
1-looming	26	12 (46%)	14 (53%)	0	0
10-looming	39	39 (100%)	18 (46%)	5 (13%)	0
10-loom (no accl*)	13	2 (15%)	2 (15%)	9 (69%)	1 (8%)
Flip screen	6	0	0	2 (33%)	4 (66%)
White receding	25	2 (8%)	13 (52%)	13 (52%)	2 (8%)
Sweep (small)	18	0	7 (39%)	8 (44%)	4 (22%)
Sweep (large)	17	0	9 (53%)	1 (6%)	7 (41%)

Some mice showed multiple responses to a stimulus, such as freezing followed by flight. Therefore, the totals exceed 100% for some stimuli. *no accl: acclimation was not conducted the day before the experiment.

We wondered whether the darkening of the arena causes the fear responses in mice. We applied a stimulus consisting of the screen flipping from gray to dark without a moving dark edge (Fig. 1*Ac*). Two mice showed rearing (33%; Table 1), but none showed freeze or flight responses (Fig. 1*F*). These results suggest that a moving dark edge is crucial to cause the fear-evoked freeze and flight responses.

Another study (De Franceschi et al., 2016) previously showed that a sweeping dark circle evoked freeze responses in 84% of mice, whereas the looming stimulus evoked flight responses. We then conducted a sweeping dark circle stimuli of 5.7° (Fig. 1*Ad*). In our study, however, the sweeping stimulus evoked a freeze in only 39% of mice (Table 1). We increased the size of the sweeping circle to 8.5° (Fig. 1*Ae*), which evoked a freeze 53% of mice, slightly increased from the smaller stimulus (Table 1). Mice in our colony did not consistently exhibit fear-based flight or freeze in response to the sweeping stimulus.

To evoke fear responses more consistently, we exposed the mice to a 10-time repeating looming stimulus. All mice exhibited flight response (Table 1). Some of them froze first, then dashed to the shelter (flight response; Fig. 2*A*; Table 1), while others exhibited immediate flight, or rearing followed by flight (Fig. 2*B*; Table 1). The speed of the mouse movement was displayed in a heatmap (Fig. 2*C*), which reveals that all mice showed flight with a high speed before moving into the hut (light blue). Some of them dashed immediately after the looming stimulus, and others froze (pink color) or reared before the flight. These mice were acclimated 1 d before the looming experiments (10 min) and on the day of the experiments (10 min). However, if mice were acclimated only the day of looming experiments, the fear responses were not reliably elicited [10-loom (no accl.); Table 1]. Only 2/13 mice exhibited flight and freeze to a 10-looming stimulus, but others showed either rearing or no response. To elicit the fear response consistently, we conducted 2-d acclimation and used the 10-time looming stimulus to examine the underlying cellular mechanism of the looming-evoked fear responses.

### Starburst amacrine cell ablation reduced fear responses

Cellular mechanisms of looming stimulus-evoked fear responses have not been fully understood. Because a moving dark spot evoked the fear responses, the OFF channel of direction-selective ganglion cells (DSGCs) in the retina is likely to be involved (Yilmaz and Meister, 2013). Our data indicated that a dark moving edge, either looming or white-receding, evoked the freeze and flight responses (Fig. 1). Therefore, we 3examined whether the motion-detection pathway is involved in causing the fear responses by ablating retinal starburst amacrine cells (SACs), critical neurons for the retinal direction-selective pathway.

We generated a mouse line crossing ChAT-cre and diphtheria toxin receptor (DTR)-expressing lines and injected a dose of DT (3.2 or 10 ng) to exclusively ablate SACs in the retina (Hillier et al., 2017; Care et al., 2019; Kim et al., 2020). We first examined the effect of DT injection on the retinal neurons by injecting DT in one eye, and leaving another eye intact. We labeled SACs using an anti-ChAT-antibody, and labeled all retinal neurons with DAPI. In control eyes without DT injection, ON SACs were labeled in the ganglion cell layer (GCL; Fig. 3*A*, I) at a density of 1122 ± 95 cells/mm^2^ (*n* = 3 eyes). In eyes with DT injection (Fig. 3*A*, II), ON SACs were reduced to 389 ± 151 cells/mm^2^ (*n* = 4 eyes, *p* = 0.01 vs control eyes, unpaired *t* test). Remaining SACs showed normal dendritic morphology and proper placement in the IPL (data not shown). In contrast, DAPI staining did not show differences between control and DT-injected eyes in the GCL (*n* = 4 eyes each, Control: 10 522 ± 836, DT: 10 393 ± 974 cells/mm^2^, *p* = 0.9 unpaired *t* test). Similar results were obtained during the time period between 6 and 10 d after the initial DT injection. The average densities of DAPI stained neurons in the GCL in control and toxin-treated samples were similar to previous observations (Jeon et al., 1998). Therefore, we concluded that the protocol we used effectively and exclusively ablated SACs.

Because DT would be administrated by intraocular injection (Materials and Methods), we also tested whether the injection itself would disrupt the retinal neurons. We injected saline to the intraocular space of the ChAT-cre x DTR mice (*n* = 5 mice, 10 eyes). The number of ON SACs and DAPI-stained cells in the GCL did not change compared with noninjected eyes (DAPI: 9903 ± 173 cells/mm^2^, *p* = 0.52; ON SACs: 1184 ± 41 cells/mm^2^, *p* = 0.51, unpaired *t* test), confirming that the intraocular injection did not affect the retinal neurons other than SACs.

Then, we examined the behavior of the ChAT-cre x DTR mice in response to the 10-time looming stimulus before and after the DT injection. Before the DT injection, 54 ChAT-cre x DTR mice exhibited similar fear responses to C57 wild-type mice. All the mutant mice exhibited fear responses: flight, freeze, or both (Fig. 3*B*; Table 2). The flight behavior was observed after a freeze or rearing in some mice (Fig. 3*B*, pink followed by light blue). In addition, four mice showed only a freeze response to the looming stimulus (Fig. 3*B*, bottom four mice). The fear response was evoked by a looming stimulus of either 2–50° expansion (no mark) or 2–9° expansion (asterisk). The saline intraocular injection did not change the fear response. All of the saline-injected mice exhibited flight behavior, which was preceded by freeze or rearing in some mice (Table 2; Fig. 3*C*).

**Table 2:** The behavioral responses to 10-time looming before and after saline and DT injections in CHAT-DTR mice

	Mouse number	Flight	Freeze	Rearing	No response
Preinjection	54	50 (93%)	29 (54%)	9 (17%)	0
Saline	5	5 (100%)	1 (20%)	1 (20%)	0
Group 1	26	22 (85%)	14 (54%)	9 (35%)	0
Group 2	23	0	0	9 (39%)	14 (61%)

Some mice showed multiple responses to a stimulus, such as freezing followed by flight, and the totals exceed 100%.

The DT intraocular injection changed the looming-evoked responses in half of the mutant mice. We divided them into two groups: fear response preserved (Group 1) and fear response disappeared (Group 2). Although DT was injected, the Group 1 mice still showed a fear response similar to the preinjected mice: flight, freeze, or both (Fig. 3*D*; Table 2). Some mice exhibited the freeze or rearing followed by flight, and four mice showed only a freeze response. In contrast, the Group 2 mice did not show either flight or freeze, but rearing was observed in 39% of mice (Fig. 3*E*; Table 2). The rest of the mice did not respond to the looming stimulus.

We initially injected 3.2 ng of DT per eye, and observed that a subset of mice exhibited no looming-evoked fear responses (Group 2; *n* = 23), but others exhibited the fear responses (Group 1; *n* = 26). Then, we increased the DT dose to 10 ng per eye, and all mice were categorized as Group 2. These mice exhibited a defensive response to the looming stimulus before the DT injection, which was eliminated by DT-mediated ablation of ON and OFF-SACs (Table 2; Fig. 3*E*). Because a previous publication failed to observe the SAC’s contribution to the looming behavior (Wang et al., 2021), we used the same looming stimulus (2–9° expansion, ours was primarily 2–50°) to examine whether the stimulus difference induced the different outcomes. The smaller looming stimulus still evoked the fear responses before the DT injection (Fig. 3*B*, asterisks) but the DT injection removed those responses (Fig. 3*E*, asterisks).

In addition to looking for looming-evoked fear responses, we examined other visual behaviors to determine that these mice maintained otherwise functional vision. Mice that developed cataracts or corneal opacity at the time of behavior testing were removed from further analysis. In all DT-injected mice, we observed normal pupillary light reflexes. Furthermore, we conducted the SLAG test to examine mouse vision (Gil-Pages et al., 2013). All the mice that we tested before and after DT injection showed a positive SLAG test response (*n* = 12 for pre-DT, *n* = 11 for Group 1, *n* = 14 for Group 2), demonstrated by their ability to both perceive and locate the wire cage during grasping. These experiments and observations indicate that the DT-injected mice, both Groups 1 and 2, had functional vision. However, approximately half of them did not exhibit visually-evoked fear responses to the looming stimulus after the DT injection.

### Optomotor response and cellular examinations

**Figure 4.**
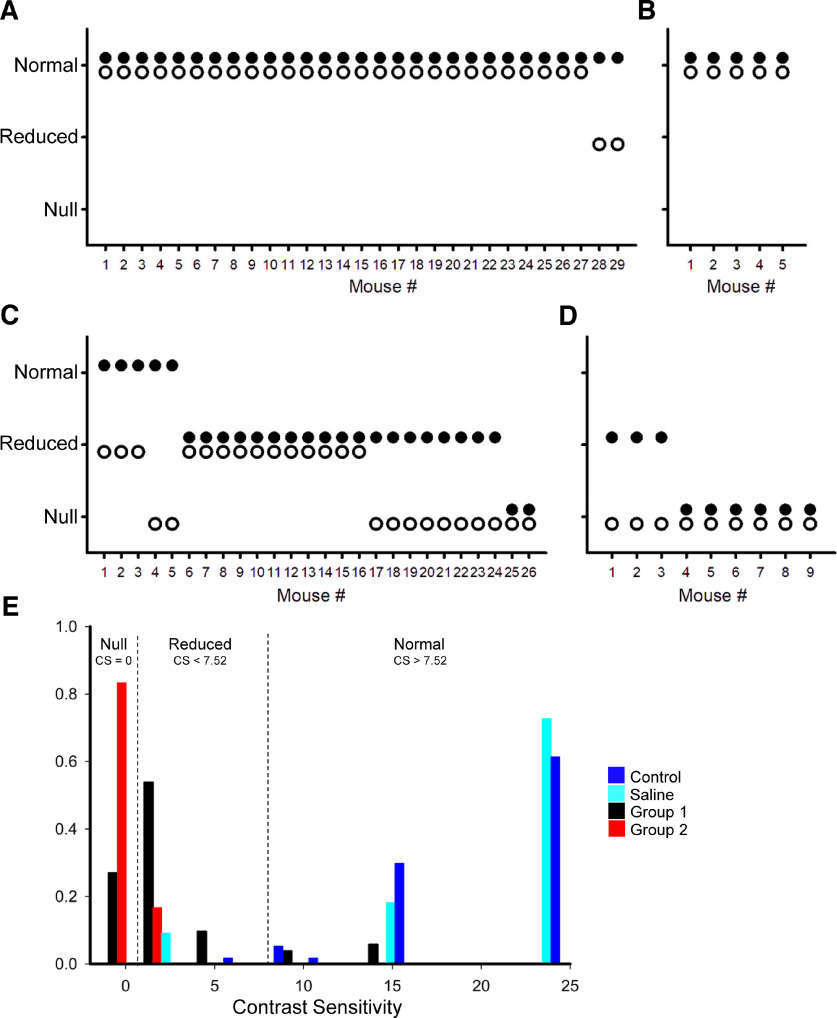
Optomotor responses (OMRs) before and after the DT injection. The black and white dots in ***A–D*** refer to one of the two eyes for each mouse. ***A***, Before the DT injection, the OMR was measured. The average contrast sensitivity was 19.1 and the SD was 5.8. When the eye exhibited within the 2 SD, it was categorized as normal. If the sensitivity was lower than 2 SD, it was categorized as reduced. If the OMR was not recognized, it was categorized as null. Most eyes before the DT injection showed normal contrast sensitivity. ***B***, Saline was injected into five mice, which all exhibited normal sensitivity. ***C***, Group 1 mice exhibited reduced or null OMRs after one week of DT intravitreal injection. For those mice, the OMR sensitivity was reduced at least one of eyes. ***D***, Group 2 mice exhibited reduced or null OMRs. These mice did not show the fear response after the DT intravitreal injection. ***E***, A bar graph showing the distribution of the contrast sensitivities for each group. The frequency was normalized to each group and is shown as the proportion of eyes with the same contrast sensitivity value per group. The contrast sensitivity values are color coded: preinjection mice (blue), saline-injected mice (cyan), Group 1 DT-injected mice (black), and Group 2 DT-injected mice (red).

The optokinetic response (OKR) disappears if SACs are eliminated (Yoshida et al., 2001), thus, measuring the OKR might indicate whether SACs were deleted by the DT injection. Because OKR is equivalent to the optomotor response (OMR) which can be conducted relatively easy in the lab (Douglas et al., 2005; Kretschmer et al., 2017), we measured the OMR from 29 preinjected mice, which showed an average contrast sensitivity of 19.1 ± 5.8 (mean ± SD). Each eye was characterized as either normal sensitivity (within 2 SD range: >7.5), reduced sensitivity (<7.5), or null OMR (no response to OMR stimulus; Fig. 4*E*). The preinjection mice exhibited normal OMR except for two eyes (Fig. 4*A*). The saline-injected mice similarly showed normal OMR (Fig. 4*B*).

The Group 1 DT-injected mice exhibited reduced OMR, indicating the decreased number of SACs (Fig. 4*C*). Some eyes even lost the OMR; however, the looming-evoked fear response still occurred. The Group 2 mice also exhibited reduced or null OMRs (Fig. 4*D*). These mice did not show the fear response to the looming stimulus. The OMR reduced or disappeared in both mouse groups (Fig. 4*E*) and no obvious distinction was recognized between them, suggesting that OMR is not an indicator for the looming-evoked fear responses.

**Figure 5.**
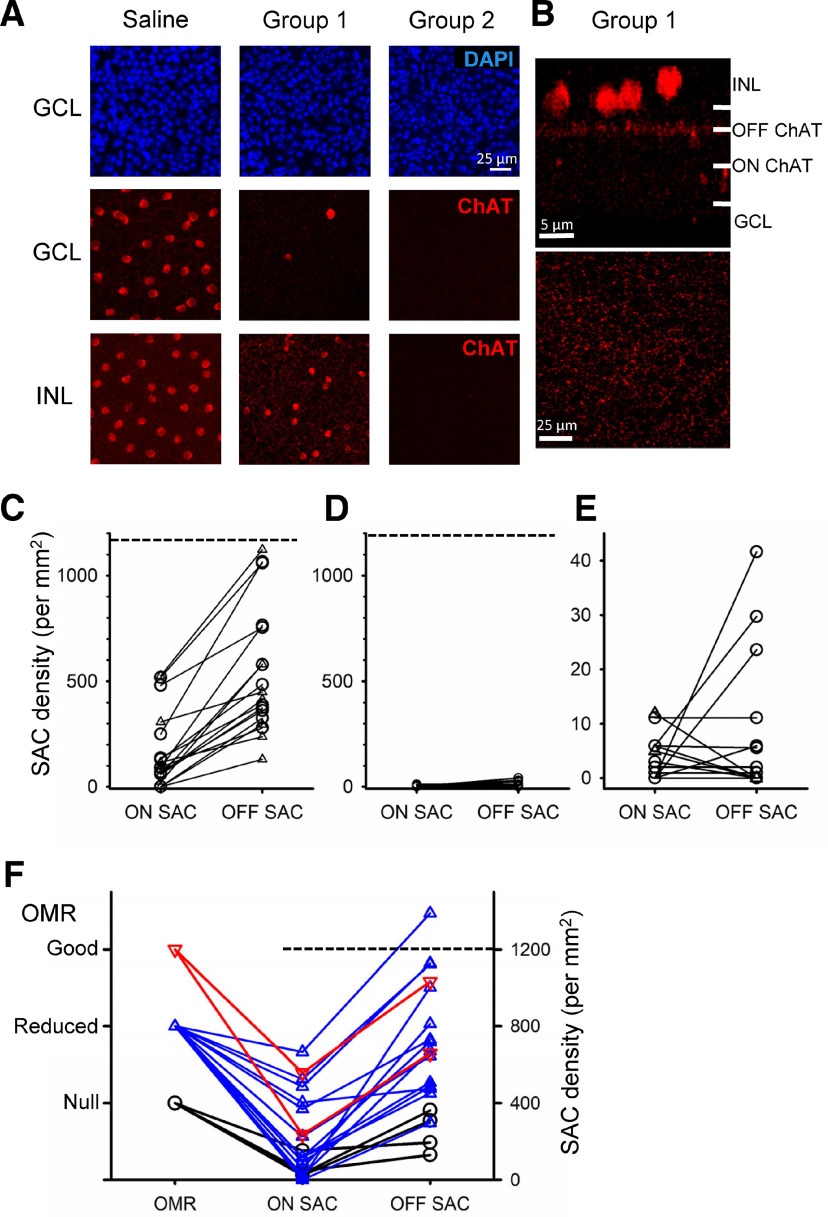
Immunohistochemical analysis of ON and OFF SACs after the DT injection. ***A***, Saline-injected eyes exhibited normal densities of GCL-layer cells, and ON and OFF SACs in GCL and INL layers, respectively. The Group 1 mice showed significantly reduced ON SACs. OFF SACs were also reduced, but still remained. In contrast, ON and OFF SACs were significantly reduced in Group 2 mice. ***B***, In Group 1 mice, the remaining OFF SACs exhibited normal dendritic structures. OFF ChAT band (side view) and dendritic network (top view) are shown. ***C***, ON and OFF SAC density in Group 1 mice. The dotted line indicates the normal density. ***D***, ON and OFF SAC density in Group 2 mice. The scale is same as the graph ***C***. ***E***, The same graph as ***D***; however, the scale was adjusted. ***F***, OMR and the densities of ON and OFF SACs were compared in twenty-one eyes from Group 1 mice. The red lines are mice that had normal OMR, blue lines are mice with reduced OMR, and black lines are mice with null OMR. Although ON SAC density was significantly decreased, some eyes exhibited OMR in normal and reduced ranges. The null-OMR mice exhibited the low ON SAC density. However, OFF SACs were relatively preserved (>20% of normal density).

To examine how surviving SACs that remain after DT injection may explain the persistence of visually-evoked fear responses, we performed immunohistochemistry on 55 retinas from 32 mice. DT-injected mice were euthanized for immunohistochemistry within 24 h after the behavioral examinations were conducted. Initially, we counted the DAPI-expressing cells in the GCL, which should include all ganglion cells and displaced amacrine cells. Based on remaining visual behaviors and lack of Cre-induced DTR expression, we expected that these neuronal populations would be unaffected by DT injection. This was supported by the number of DAPI-expressing cells, which was not different among the three mouse groups: saline-injected, Group 1, and Group 2 (*p* = 0.19, one-way ANOVA; Table 3; Fig. 5*A*). This indicated that the two doses of DT concentrations we used did not affect neural populations other than SACs.

**Table 3:** The number of cells in the GCL and SACs after saline and DT injections

	Number of eyes	DAPI (GCL) (cells/mm^2^)	ON SAC (cells/mm^2^)	OFF SAC (cells/mm^2^)
Saline	10	9825 ± 275	1184 ± 41	1244 ± 33
Group 1	29	9762 ± 227	166 ± 37*	555 ± 62*
Group 2	26	9162 ± 303	3 ± 1*^#^	9 ± 4*^#^

Values are mean ± SEM. Statistical differences are described in text. *denotes statistical significance compared with the saline group, and ^#^denotes statistical significance compared with Group 1.

Then, we counted ON and OFF SACs both in Groups 1 and 2 mice. The number of ON SACs was significantly reduced both in Groups 1 and 2 mice compared with saline-injected mice (Table 3; *p* = 10^−17^ for Group 1 and *p* = 10^−10^ for Group 2 vs control eyes, unpaired *t* test). In Group 1 mice, the average ON SACs were higher than those in Group 2 mice; however, some mice showed 0–1% of the normal cell density, and still exhibited the looming-evoked fear response (Fig. 3). The reduced-ON SACs explain the reduced OMR in Figure 4*C*,*D* (Joly et al., 2014). OFF SACs were also significantly reduced compared with the saline-injected eyes (Table 3); however, there was a difference between two groups; OFF-SACs in Group 1 were reduced by approximately half and preserved relatively normal dendritic structures (Fig. 5*B*), whereas OFF-SACs in Group 2 mice were reduced to ∼1%. The number of remaining OFF SACs was significantly different between two groups (*p* = 10^−9^ Group 2 vs Group 1, unpaired test). ON SACs appeared to be more effectively ablated than OFF SACs (Fig. 5*B–E*), most likely attributable to the difficulty of DT penetration to the INL compared with the GCL from the intravitreal injection site. Because the looming-evoked fear response disappeared in the Group 2 mice, these results suggest that OFF SACs contribute to the initiation of fear responses.

We further examined the relation between OMR and the SAC numbers using a subset of Group 1 mice (*n* = 21 eyes; Fig. 5*F*). As stated in the previous section, ON SACs were reduced in these mice but OFF SACs were still preserved. Some eyes showed OMR within the normal range and reduced range (Fig. 5*F*, red and blue lines). Five eyes showed no OMR, of which ON SACs were reduced to 55 ± 25 cells/mm^2^ (range 20–153, ∼5% of the normal density), whereas the density of OFF SACs was 243 ± 42 cells/mm^2^ (20% of the normal density) and the looming stimulus evoked freeze or flight responses. In contrast, the OFF-SAC density was reduced to ∼1% in Group 2 mice (Table 3), with ON-SAC density being similar to the ∼5% remaining ON-SAC population in Group 1, which did not show a fear response to the looming stimulus. Taken together, these results indicate that OFF SACs are required for the looming-evoked fear response.

## Discussion

In the present study, we found that the fear responses, flight and freeze, were consistently evoked by a 10-time looming stimulus with 2-d acclimation. We examined whether SACs were involved in the neural pathway for looming-evoked fear responses by injecting DT into the intravitreal space to ablate those cells. The fear responses disappeared in approximately half the DT injected mice. The reduced OMR did not correlate with the disappearance of the fear responses, and ON-SACs were effectively ablated by DT-injection. This suggests that the contribution of ON SACs to looming is low, but does not directly rule out the possibility of their involvement. The immunohistochemistry data revealed that mice with a considerable number of surviving OFF SACs maintained fear-evoked responses, whereas the fear response disappeared from mice where OFF SACs were almost entirely eliminated by DT. These results indicate that OFF SACs and downstream neurons, including the motion detection pathway, are critical for looming-evoked fear responses.

### Looming activating pathway in the CNS

The looming stimulus evokes freeze and flight responses, which serve to defend mice from a perceived threat. Threats can be categorized as imminent in the form of circa-strike and postencounter with an anticipation of a possible nociceptive event (Mobbs et al., 2009). The circa-strike is characterized by a direct predator attack, which may evoke panic behaviors in prey. In contrast, postencounter is defined as higher-level processing involving monitoring and predicting the outcome with a slower time course. The looming and sweeping stimuli mimic the imminent threat and postencounter, respectively.

Activity loci have been investigated in the human brain using the electroencephalogram (EEG) and fMRI. Imminent threats increase activity in the midbrain, including the periaqueductal gray (PAG), whereas postencounter threats activate the forebrain, hippocampus, hypothalamus, and amygdala (Mobbs et al., 2009). Alternatively, connections among the amygdala, hypothalamus, and PAG receive threat signals and produce defensive behavior (Adolphs, 2013). The amygdala is a well-known center for fear, which is activated by a looming visual cue but not by a receding stimulus (Cappe et al., 2012). The amygdala responds strongly to frightened human faces and threatening animals, such as tarantulas, rather than neutral faces, animals, and objects (J. Yang et al., 2012). The PAG in the midbrain is also sensitive to approaching threats compared with receding threats or neutral objects (Coker-Appiah et al., 2013).

The neural pathway to the amygdala on detection of the looming visual stimulus has been investigated using mouse models. The parvalbumin-positive (PV^+^) excitatory neurons in the mouse superior colliculus (SC) are sensitive to the looming stimulus, which projects to the parabigeminal nucleus (PBGN) and lateral posterior thalamic nucleus (LPTN), which relay inputs to the amygdala (Shang et al., 2015, 2018; Wei et al., 2015). Interestingly, PV^+^ neuron projection through the PBGN evokes flight and freeze, whereas the neurons projecting through the LPTN induce only freezing behavior (Shang et al., 2018). A recent study revealed another critical projection from the mouse SC to the amygdala. A neural projection from SC to the ventral tegmental area in the midbrain, then to the central nucleus of the amygdala, is crucial for looming stimulus-evoked flight behavior (Zhou et al., 2019). It has not been fully understood how these separate pathways control looming-evoked behavior. However, multiple investigations assured that the looming stimulus activates the neural projection from SC to the amygdala that evokes fear responses.

### Looming-evoked pathway in the retina

It is certain that the looming stimulus activates retinal neurons before the SC and amygdala. However, we have just begun to examine the retinal neural pathway for looming detection. In the fly eyes, the lobula plate/lobula columnar, Type II (LPLC2) neuron has been identified as an ultraselective looming detector (Klapoetke et al., 2017). The lobula plate is analogous to the vertebrate colliculus (Sanes and Zipursky, 2010), and the LPLC2 cells are neurons distinct from directionally-selective cells. These cells strongly respond to a looming stimulus, but weakly to a moving stimulus in a lateral direction. Similarly, the lobula giant movement detector (LGMD) and its downstream neurons are designated looming detectors in the locust eyes (Gabbiani et al., 2002; Guest and Gray, 2006).

In the mouse retina, a large number of ganglion and amacrine cell types have been recently reported (Baden et al., 2016; Yan et al., 2020). Because these cells encode image features, there might be a looming detector similar to the fly and locust eyes. Alternatively, multiple types of direction-selective cells, which respond to specific directions of motion, may detect the looming stimulus, which activates the SC-amygdala pathway to evoke fear responses.

Yilmaz and Meister (2013) predicted that the looming behavior is induced by a transient OFF channel, such as the PV-5 cells, ON-OFF direction-selective ganglion cells (DSGCs), and W-3 cells. Münch et al. (2009) examined the looming stimulus-evoked responses in PV-5 cells, which are OFF-responding, large ganglion cells. They strongly responded to an approaching motion, suggesting that these are the looming detector. However, unlike the looming detector in the insect eyes, the receptive field of PV-5 cells was not large. Also, behavioral testing has not been conducted with PV-5 cell-disrupted mice. PV-5 cells are potentially the same as the transient OFF- α ganglion cells in the next paragraph (Farrow et al., 2013), and it is inconclusive whether the PV-5 cells are the looming detector in the mouse retina.

Kim et al. (2020) recently reported that the vGluT3 amacrine cells are the looming detector. vGluT3 cells are a unique type of amacrine cell, which release glutamate as a neurotransmitter onto W-3 and OFF-α ganglion cells (Lee et al., 2014, 2016). When the vGluT3 cells transiently responded to a looming stimulus, the W-3 ganglion cells similarly responded in a transient fashion, and the OFF-α ganglion cells exhibited an increasing response depending on the stimulus expansion speed. This result indicates that the W-3 ganglion cells code the onset of the looming stimulus, while the OFF-α cells encode the speed of expanding motion (Kim et al., 2020). Remarkably, when vGluT3 amacrine cells were removed by diphtheria toxin, the freezing response to a looming stimulus was significantly reduced. Therefore, vGluT3 amacrine cells serve as a looming detector. The involvement of the OFF-t α ganglion cells in the looming response was confirmed by genetic methods by Wang et al. (2021).

We observed that fear responses were evoked by looming and white receding stimuli, but not by the flip screen, indicating that the moving dark edge is critical. Therefore, we targeted the SACs using diphtheria toxin (DT) for ablation, as SACs provide synaptic inputs to DSGCs and are crucial for motion detection in the retina (Yoshida et al., 2001). The DT injection did not always completely remove SACs, attributable to the conservative DT dose we injected. However, the varied SAC reductions and behavior outcomes offered a clue for the underlying cellular mechanism. When the DT ablated primarily ON SACs, we observed the reduced OMR; however, the looming-evoked fear responses still occurred (Group 1). When both ON and OFF SACs were eliminated, the fear responses were also removed (Group 2). The number of DAPI-stained GCL cells did not change in both groups, indicating that other neurons were not affected by the DT injection. While we cannot directly rule out there being any impact from ON SACs, these results indicate that OFF SACs and downstream direction-selective ganglion cells (DSGCs) are crucial for looming visual stimuli-evoked flight.

How do our results complement the results of Kim et al. (2020) and Wang et al. (2021)? The SAC-DSGC and vGluT3-W3/off-α connections are two separate neural pathways in the retina. Both SACs and vGluT3 are amacrine cells, and there are no synaptic connections among them (Lee et al., 2014). Kim et al. (2020) found that the vGluT3 removal suppressed the freeze response to the looming stimulus. In contrast, we found that the SAC disruption eliminated both flight and freeze responses. Potentially, two separate retinal circuits mediate different aspects of looming behavior, similar to the multiple SC-amygdala pathways (Shang et al., 2018). Alternatively, two separate pathways may coordinate to sense looming and generate a fear response. The W-3 ganglion cells possess a large antagonistic surround region and sense local motion (Kim and Kerschensteiner, 2017). ON-OFF DSGCs sense high-speed motion (Sivyer et al., 2010). When one of them is disrupted, mice may no longer respond to a looming stimulus.

Wang et al. (2021) examined the effect of SAC ablation using the same methods we did, but did not observe the reduced looming-triggered defensive response. We postulated a couple of factors led to the different outcomes: stimulus size and SAC density. We matched their looming stimulus by changing the expansion to 9° instead of the 50° we used. However, the DT injection in our mice removed the defensive response, indicating that stimulus differences may not be the reason for the different looming responses. Another possibility is that they showed reduced density of SAC in their supplement figure but were not clear about the ON versus OFF SACs. In case they only examined ON SACs, OFF SACs might still have existed and induce the defensive response, similar to our Group 1 mice.

In conclusion, we found that OFF SACs and downstream pathways are crucial for looming detection and driving optically-derived fear responses. However, because we could not eliminate ON and OFF SACs separately, potential contribution of ON SACs to the looming response cannot be ruled out. Multiple neural pathways in the retina might be critical to inducing the defensive response to an approaching shadow. We believe that our study has revealed a link between early visual processing in the retina by a specific cell type and visual cue-evoked behavioral outcomes.

## Synthesis

Reviewing Editor: Nicholas J. Priebe, University of Texas at Austin Decisions are customarily a result of the Reviewing Editor and the peer reviewers coming together and discussing their recommendations until a consensus is reached. When revisions are invited, a fact-based synthesis statement explaining their decision and outlining what is needed to prepare a revision will be listed below. The following reviewer(s) agreed to reveal their identity: NONE.

Two reviewers and the reviewing editor evaluated your revision of the manuscript “OFF Starburst Amacrine Cells in the Retina Trigger Looming-evoked Fear Responses in Mice.” We all agree that this manuscript demonstrates an important role for OFF starburst amacrine cells in generating the fear-evoked responses elicited by a looming stimulus. There was consensus, however, that the role of the ON Starburst amacrine cells remains unclear and that these cells could contribute to a looming response. The authors write that “these results indicate that OFF SACs are required for the looming-evoked fear response” (line 380) and “that OFF SACs and downstream neurons, including the motion detection pathway, are critical for looming-evoked fear responses” (line 390-391). All agree that there should be clarification that it remains unclear whether, or how, ON starburst amacrine cells might contribute to looming-evoked fear behaviors.

## Author Response

SYNTHESIS

Two reviewers evaluated the manuscript “OFF Starburst Amacrine Cells in the Retina Trigger Looming-evoked Fear Responses in Mice” and both reviewers agree that the authors provide evidence that OFF amacrine neurons are sufficient to trigger escape behavior. There was consensus, both in the reviews and in the consultation that followed, that while there is a demonstration that OFF amacrine cell contribute to escape behavior, ON amacrine cells may also contribute. Both reviewers felt that the conclusion that OFF SACs are critical for looming evoked fear responses was not supported by the data presented. The reviewers also raised questions about how the present findings fit with previous work examining the effects of SAC ablation and fear responses. These issues and others are detailed below, which you should find useful in revising your manuscript, if you decide to resubmit it to eNeuro or to another journal.

Major Issues:

1) Are OFF SACs critical for looming-evoked fear responses?

A major conclusion of the manuscript is that OFF SACs are essential for looming-evoke fear responses, based on the effects of ablating subsets of SACs. But, the data show instead that OFF SACs are sufficient to drive the fear response. No experiments were done to test if ON SACs alone could similarly drive the fear response. The authors conclude that OFF starbursts as ‘special components’ underlying the fear response but they may be no different from the ON SACs in terms of their contribution.

The difference in density of ON SACs and OFF SACs is less than 1% (3 vs 9 cells/mm2 compared to ∼1200cells/mm2 for control) for Group 2. Since most SACs are ablated in Group 2, interpreting changes in behavior from cell groups with less than 1% difference in density, seems premature. In fact, the median ON SAC density is less than the OFF SAC density (Fig. 5D). This could be potentially be tested by preferentially ablating the OFF SACS and maintaining ON SACs through titrated subretinal injection with diphtheria toxin. Without such an experiment it is not appropriate to designate OFF SACs (and implicitly not ON SACs) as performing a specific role in the looming response.

We would like to restate our logic underlying the group 1 and 2 mice and looming-evoked fear responses. In group 1 mice, the intraocular diphtheria toxin (DT) injection significantly reduced the ON SACs (Figure 5A-B). In some mice, ON SACs were 0 to 1% of the normal range. However, looming-evoked fear responses still occurred (Figure 3D). In these mice, OFF SACs were relatively preserved, probably attributable to their location deep into the retinal tissue. When we increased the DT dose and ablated both ON and OFF SACs (Figure 5C), looming evoked fear responses disappeared (Figure 3E). Because the difference between group 1 and 2 mice is the number of remaining OFF SACs, we concluded that OFF SACs are responsible for the looming-evoked fear response. As the Reviewer pointed out, it would be best to investigate the looming-evoked responses with mice where only OFF SACs but not ON SACs are ablated. However, the proposed experiment cannot be easily accomplished because ON and OFF SACs share similar genetic backgrounds (Yan et al., 2020). Although they express distinct transcriptional factors at the developmental stage, no distinction has been detected after P18-P19. Currently, no genetic tools are available 2 to target OFF SACs exclusively. Therefore, using the subretinal injection to delete predominantly OFF SACs might work. Based on the Reviewer‘s suggestion, we have conducted the subretinal injection method to target OFF SACs. We used the ChAT-cre mice (P1-P5), and conducted subretinal injection with the AAV2-FLEX-DTR-GFP (Addgene #124364). We used P1-5 pups for the subretinal injection, expecting that the viral vector would spread to a larger proportion of the retina. We targeted the ventral retina, where the superior visual field projects to. We anticipated that many OFF SACs in the ventral retina but not ON SACs would express DTRs. After more than a month, we injected DT into the intravitreal space and examined the looming-evoked behavior. The subretinal injection is a challenging procedure. We injected 15 ChAT-cre pups, but only two pups still exhibited normal pupillary light reflexes. Eyes in other mice showed injury or weak pupillary light reflex. After the DT injection, the two mice still showed a freeze response to a looming stimulus. We examined the retinal tissue and found that both ON and OFF SACs reduced but still remained. There was no reduced number difference between ON and OFF SACs (xx). Therefore, the subretinal injection method does not work for OFF SACs targeting ablation.

Our conclusions are same as our previous manuscript; OFF SACs and the downstream pathway are critical components for looming-induced fear responses. Our conclusion does not state that OFF SACs exclusively cause the looming-evoked response because other retinal neurons, such as vGluT3 amacrine cells/tOFF ganglion cells and PV5 OFF ganglion cells (publications cited by our manuscript) are also important for looming-evoked fear responses.

We revised the description of our logic in the Results section (page 19) to clarify our point. Although we could not independently ablate OFF SACs, our data revealed OFF SACs’ function as a looming mediator.

The figure below shows retinal ON and OFF SAC images after subretinal AAV-DTR injection and subsequent DT injection. SACs were reduced only in mouse 1 right eye; however, both ON and OFF layers were similarly reduced. SACs in other eyes were relatively normal, suggesting that AAV-DTR injected in the subretinal space did not spread out.

1 Left ON 1 Left OFF

1 Right ON 1 Right OFF Ms 1 left Ms 1 right Ms 2 left Ms 2 right SAC Density (Per mm2 ) ON SAC OFF SAC 0 200 400 600 800 1000 1200 3

2) How may the current results be reconciled with previous ablation experiments?

Nearly 100% SACs were ablated in the Wang, 2021 study, yet they did not find a change in fear response to 9deg and 36deg spot stimulus. Kim et al, 2002, used spots ranging from 1deg to 17deg to trigger fear response in mice. If SACs are involved in fear response and fear response is generated only by large spots, then why in the presence of SACs were fear responses observed with spots of smaller sizes? Showing the dependence of onset and saturation of fear response on the spot size, for control and DT injected mice might be helpful.

We mainly used looming stimuli of 2-50 deg. Additionally, we used the 2-9 deg looming stimulus to match the experiment of Wang 2021. In our results, both stimuli induced fear responses and DT injection removed the response (Figure 3 marked with asterisks). Therefore, the differential data, regarding the DT injection to the SAC ablations, between Wang et al. and us was not attributable to the stimulus difference. Wang et al. 2021 did not show the DT effect clearly on both ON and OFF SACs. There was also no description regarding ON and OFF SACs. We assumed that they did not observe the OFF SACs specifically. If the OFF SACs were partially preserved in Wang’s data, this would be similar to our group 1 mice that showed the looming-evoked fear response. We have described this in the Discussion (pages 25-26).

3) Differential responses to dark and bright stimuli The fact that bright expanding spot doesn’t yield the same degree of fear response as a dark expanding spot in control mice, suggests that asymmetry of ON and OFF pathway signaling could be mediating these disparate behaviors (see Chen et al. 2016). Thus, with a large fraction of SACs deleted, it is even more difficult to distinguish these behaviors solely from the standpoint of cell count. These concerns are not properly addressed or discussed in the paper.

To distinguish the impact of surviving ON and OFF SACs, I suggest a simple test: Measure the OMR of control and DT injected mice to moving bright bar and dark bar respectively in the same direction. Presumably the OMRs in control mice to bright and dark bars are indistinguishable. If the numerical difference between surviving ON and OFF SAC cells is sufficient for producing different OMR to bright and dark moving bars in DT injected mice, then it serves as a validation that the density difference between residual SACs could preferentially influence detection of dark expanding spots.

In our experiments, we did not use the bright expanding spot as the reviewer described. We instead used the white receding circle stimulus, in which dark edge moves from the peripheral to the center of the monitor (Figure 1Ab). We examined whether the dark edge moving direction either from center to the periphery or periphery to center is important to induce the fear responses. The white receding stimulus induced freezing in approximately half the mice tested. In contrast, the looming stimulus with the dark expanding circle induced reliable fear response (Figure 2-3). Therefore, we used the looming stimulus to pursue the following experiment, and did not use the other stimuli.

We have conducted the OMR recordings with alternating white and black bars in the same direction with the control and DT injected mice, as the reviewer suggested (Figure 4). Even though the OMR diminished in some mice, they still exhibited the looming-evoked fear response. We have revised the description of how the OMR testing was conducted in the Method section to clarify the experiment. 4 4) How does contrast sensitivity vary across different groups of animals?

If contrast sensitivity outside 2SD is considered reduced sensitivity what is the threshold in units of SD for null OMR? The results in Figure 4 do not provide clarity on how sensitivity varies within groups across mice and across groups. Showing the actual distribution of contrast sensitivity at the optimal spatial frequency for different mice and marking the boundaries for normal, reduced and null OMR would be helpful.

When the mouse does not respond to the stimulus, the OMR is considered null and the contrast sensitivity would be 0. We did not designate null OMR based on number of SD from the mean. We revised Figure 4 and added a new panel to show the exact contrast sensitivities are shown in a distribution graph that includes the proportion of eyes from each group (control, saline injected, and DT-injected groups) that demonstrated each contrast sensitivity value. The graph includes the boundaries for what is considered normal, reduced, and null.

5) The heat map of speed in Figure 2 is a nice way to show the different types (freeze, flight, rear) and time onsets of responses across animals. However, the sequence of response and time when each response occurs, are likely to have some degree of variability across different presentation of the same stimulus. Are the speeds in Fig. 2C averaged across all trials for each animal or a single trial selected for each animal? What is the trial-to-trial variability in response of an animal? If the response variability is high, then it would mean that responses are driven not just by loom stimulus but also by some internal physiological state. Figure 2C shows mouse speed before and during the looming stimulus. Each row indicates speed of one mouse over the time without averaging any points or between mice. We acclimated each mouse one day before the experiment, and thus, we tested the looming stimulus only once for each mouse. We do not have data for the trial-to-trial variability for a single mouse. Some mice exhibited flight immediately after the looming stimulus started, while others froze first, then flight seconds later. This type of variability has been described in the papers from Yilmaz and Meister (2013), and De Franceschi et al (2016). We tested 51 DTR mice and all exhibited flight, freeze, or both, and thus, we believe that the looming-evoked response was robust and consistent.

Minor Issues:

1) Why were those 2 specific doses of DT used in the study? Was the lower dose meant to retain a certain fraction of SACs to distinguish behavioral effects compared to complete ablation?

We used the lower dose DT initially and observed that the effect was variable in both group 1 and 2. We later increased the DT dose, which induced consistent results (group 2 mice). 2. Fig 5D: Are there specific behavioral differences between mice that have significantly higher surviving OFF SACs relative to ON SACs, compared to mice that have similar densities of surviving ON and OFF SACs?

We have not noticed behavioral differences between these two groups of mice, other than the visual behavior shown in our manuscript that the higher remaining OFF SAC mice have strong 5 looming response while the mice with >90% ablation in both populations have no looming response. All of our SAC ablated mice had normal pupillary light reflexes and positive SLAG tests, indicating the normal vision. Our data are consistent with previous works in which behavioral differences between control and SAC deleted mice have not been reported (Yoshida et al., 2001; Hillier et al., 2017).

3. Statistical tests of significance, especially between dose and control groups are largely missing. These need to be performed and added to the figure. In the results section, we included the statistical tests of significance that we performed to compare the DAPI cells, ON SACs, and OFF SACS each to the control. To make this clearer, we have added asterisks to Table 3 to denote statistical differences between the cell numbers.

4. Methods: The authors should provide description of the staircase algorithm or include a reference for it. The manufacturer’s name is missing and needs to be added. The contrast range and size of contrast steps also needs to be included because the behavioral outcomes critically depend on these parameters.

The methods section has been edited to provide a clearer description of what was used. The manufacturer’s name was mentioned earlier in the paragraph, but we have added it once more next to the staircase algorithm description as well as a reference to Prusky et al. (2004) which uses the same staircase algorithm.

5. What is the luminance level of the arena where the mouse was performing task?

The light level used for the arena was 7 × 105 photons/μm2 /s as described previously (Koehler et al., 2019). We have updated the methods section to clarity of this.

6. Is the morphology of the surviving SACs impacted after DT injection? The authors should consider showing en face images of surviving SAC dendrites from immunohistological labeling. The dendritic morphology of surviving SACs was similar to those from normal SACs, although the number of cells/processes reduced. The remaining SACs still form OFF ChAT bands, which we now show in the additional panels in Figure 5.

7. Do the line colors in Fig. 1C-E correspond to a particular mouse being tracked for each stimulus condition? This needs to be explained. Alternatively, monochrome color can be used if identity of mouse is not tracked. It needs to be clarified why different sample size is used for loom vs receding circle vs flip screen. No, the line colors do not correspond to a particular mouse being tracked. Different colors were used so that the lines on the graph do not merge together and look like fewer lines. We edited Figure 1 so that each graph uses a different set of colors for each one to clarify that it is not the same mice being tested for each condition. We also edited the figure legends to reflect this change. Each mouse was only tested using one of the three stimuli (looming, receding circle, or flip screen). We found that acclimation was important for inducing the responses, so we only 6 tested one condition per mouse to more reliably see the effect of the chosen stimulus. The methods section has been updated to reflect this.

8. Is there an explanation for selecting different sample size in Fig. 1 E (n=13) and Fig. 1F (n=6)? If data were collected for a subset of mice, were they randomly selected from the full cohort? As mentioned above, each mouse was only tested using one of the three stimuli (looming, receding circle, or flip screen). It was not the same group of mice being tested for each condition because they needed to be acclimated the day before testing as well as the day of testing. Because we were looking at fear responses, we did not want the mice to acclimate to the different stimuli and not produce a response. The methods section has been updated to clarify this point.

9. The authors mention OFF DSGCs are likely involved in fear responses to loom (Page 14).

The author should correct this, because there has been no conclusive evidence showing existence of OFF DSGCs in the mouse retina.

The description is from the paper cited (Yilmaz and Meister, 2013) and we wanted to say that the OFF channel of DSGCs are likely involved. This might be the OFF DSGCs or the OFF channel of ON-OFF DSGCs. To avoid the confusion, we have revised the description.
